# Cecil Augustus Joll

**Published:** 1945

**Authors:** 


### CECIL AUGUSTUS JOLL, M.D., B.S., B.Sc., F.R.C.S.

A BRISTOL-BORN surgeon with an international reputation has recently passed away in Mr. Cecil Augustus Joll. He was a son of the late Mr. W. F. Joll, dentist, who practised in Bristol for many years.

Educated at University College, Bristol, University College Hospital, London, and London Hospital, and also in Paris. He took the M.B., B.S. (London) degrees with honours in medicine, surgery, pathology and forensic medicine, and hygiene, and won a gold medal. He was also M.D., B.Sc. (Bristol), B.Sc. (London), and F.R.C.S.

He was Senior Surgeon to the Royal Free Hospital, London, and Lecturer on Surgery ; Senior Surgeon to the Miller Hospital, Senior Surgeon to the Royal Cancer Hospital, and Hunterian Professor, Royal College of Surgeons, 1923 and 1939.

Mr. Joll wrote numerous books and articles on medical and surgical subjects.

He leaves a widow, a son, and a daughter.

